# Multimodal Intervention to Improve Functional Status in Hypertensive Older Adults: A Pilot Randomized Controlled Trial

**DOI:** 10.3390/jcm8020196

**Published:** 2019-02-06

**Authors:** Liliana C. Baptista, Byron C. Jaeger, Stephen D. Anton, Anthony A. Bavry, Eileen M. Handberg, Anna K. Gardner, Sara A. Harper, Lisa M. Roberts, Bhanuprasad Sandesara, Christy S. Carter, Thomas W. Buford

**Affiliations:** 1 Department of Medicine, Division of Gerontology, Geriatrics and Palliative Care, University of Alabama at Birmingham, Birmingham, AL 35205, USA; lbaptista@uabmc.edu (L.C.B.); saharper@uabmc.edu (S.A.H.); lmroberts@uabmc.edu (L.M.R.); cartercs@uabmc.edu (C.S.C.); 2 Center for Exercise Medicine, University of Alabama at Birmingham, Birmingham, AL 35205, USA; 3 Department of Biostatistics, University of Alabama at Birmingham, Birmingham, AL 35294, USA; bcjaeger@uab.edu; 4 Department of Aging and Geriatric Research, College of Medicine, Division of Clinical Research, University of Florida, Gainesville, FL 32610, USA; santon@ufl.edu (S.D.A.); akgardner@ufl.edu (A.K.G.); bsandesara@ufl.edu (B.S.); 5 Department of Medicine, College of Medicine, Division of Cardiovascular Medicine, University of Florida, Gainesville, FL 32610, USA; anthony.bavry@va.gov (A.A.B.); eileen.handberg@medicine.ufl.edu (E.M.H.); 6 North Florida/South Georgia Veterans Health System, Gainesville, FL 32608, USA

**Keywords:** angiotensin-converting enzyme inhibitors, antihypertensive medication, exercise, functional status, hypertension, aging

## Abstract

This pilot randomized controlled trial (RCT) was designed to provide the preliminary data necessary to conduct a full-scale trial to compare the efficacy of differing first-line antihypertensive medications in improving functional status in older adults, when combined with exercise. The primary objectives were to assess study feasibility, safety, and protocol integrity. Dependent outcomes included gait speed, exercise capacity, body composition, and systemic cardiometabolic biomarkers. Thirty-one physically inactive older adults (70.6 ± 6.1 years) with hypertension and functional limitations were randomly assigned to (1) Perindopril (8 mg/day *n* = 10), (2) Losartan (100 mg/day; *n* = 13), or (3) Hydrochlorothiazide (HCTZ: 25 mg/day; *n* = 8). Participants were also assigned to a 24-week multimodal exercise intervention, separated into an aerobic and concurrent (aerobic + resistance) phase to evaluate potential mode effects. Retention was 84% (26/31), and compliance was >90% and >79% with medication and exercise, respectively. A total of 29 adverse events (Perindopril = 5; Losartan = 12; HCTZ = 11) and one unrelated serious adverse event were observed throughout the trial. Overall, this pilot RCT provided critical data and identified several challenges to ultimately designing and implementing a fully powered trial.

## 1. Introduction

Functional status is an indicator of health and health-related quality of life [1], and the loss of physical function in advanced age is associated with the onset of disability, the loss of independence, and increased risk of cardiovascular morbidity and mortality [2,3,4,5,6,7]. For instance, the decline in usual-pace gait speed—a known measure of functional status [8,9]—has been associated with the incidence of stroke and adverse events after cardiac surgery [5], as well as with cardiovascular and all-cause mortality [2,4,7].

Evidence from the Charleston Heart, Cardiovascular Health, Three-City, and Lifestyle Interventions and Independence for Elders studies [10,11,12,13] has indicated that compared to normotensive peers, hypertensive seniors experienced accelerated declines in gait speed and higher rates of disability. Thus, older adults with hypertension represent a specific high-risk group for accelerated functional decline and cardiovascular-related events [10,11,12,14,15]. Given the remarkable demographic shift in the older population in the next decades [16], along with the increase of hypertension and cardiovascular disease prevalence [17] and the disproportionate utilization discrepancy of healthcare resources by the elderly [18,19], there is a need to identify effective interventions that minimize the clinical and economic effects associated with age-related functional decline.

As previously outlined [15,20], accumulating evidence has suggested that first-line antihypertensive medications may have an important impact on the rate of functional decline among older adults with hypertension. In particular, angiotensin-converting enzyme (ACE) inhibitors have been suggested as a promising therapeutic option [21,22,23] due to their blood pressure regulation mechanisms [24] and to their potential independent pleiotropic cardiovascular effects [25,26]. Notably, it appears that the impact of antihypertensive medications may be strongest among persons engaging in physical exercise. Prior work [13,22,27,28,29] has suggested that the choice of antihypertensive medications may impact functional status when combined with exercise [18,30], though disparate findings exist [31]. Therefore, as previously outlined [15], the primary objective of this pilot randomized controlled trial (RCT) was to evaluate feasibility, safety, and protocol integrity to compare the efficacy of different first-line antihypertensive medications on physical function in older adults, when combined with physical exercise. These preliminary data will provide evidence to support the conduct of a full-powered RCT to test our central hypothesis that, compared to other antihypertensive medications, ACE inhibitors improve functional status of hypertensive seniors, when combined with regular exercise.

## 2. Materials and Methods

### 2.1. Study Design and Procedures

The study design has been described in detail previously [15]. Briefly, the study was a three-arm, pilot RCT conducted in Gainesville, Florida (USA), between 2013 and 2017. Participant safety was overseen by a comprehensive study team, including the principal investigator, study physicians, study staff, and an appointed Data Safety Monitoring Board. All statistical analyses were performed by a biostatistician who remained masked to the intervention assigned until the completion of analyses, providing a triple-masked design. Prior to enrollment, all participants provided written informed consent approved by the University of Florida Institutional Review Board. Additionally, the study was registered at www.clinicaltrials.gov (NCT01891513).

### 2.2. Participants

Community-dwelling adults with hypertension were recruited from the Gainesville, Florida, community using a multimodal approach of direct mailings, newspaper advertisements, and other community approaches. Eligibility criteria included (1) age ≥60 years; (2) untreated systolic/diastolic blood pressure (SBP/DBP) ≥140/90 mmHg or treated with antihypertensive medication consistent with the *Seventh Report of the Joint National Committee on Prevention, Detection, Evaluation, and Treatment of High Blood Pressure* guidelines at the beginning of the study [32]; (3) an inactive lifestyle, defined as <150 min/week of moderate physical activity [33], assessed by the Community Health Activities Model Program for Seniors (CHAMPS) questionnaire [34]; (4) objective signs of functional limitations, defined as walking speed <1.2 m/s during a 400-m usual-paced test [35]; and (5) willingness to participate in all study procedures.

Exclusion criteria included a primary indication of ACE inhibitor use, known hypersensitivity to ACE inhibitors, treatment with ≥3 antihypertensive drugs, or uncontrolled hypertension (treated office blood pressure >180/110 mmHg). Additional exclusion criteria can be seen in the Supplemental Materials, Table S1.

### 2.3. Randomization

Randomization and dispensing of study medication were conducted by an academic investigational pharmacy. Block randomization stratified by gender was used to assign subjects to intervention arms, with a 1:1:1 allocation ratio, to ensure approximately equal accrual to each intervention group throughout the study. The randomization scheme was performed by an independent biostatistician using a random number generator program specifically designed for this purpose (Mersenne-Twister) and was sent directly to the academic investigational pharmacy (University of Florida Investigational Drug Service). Treatment allocation was concealed from all involved (investigators, study staff, and participants) until the end of the study.

### 2.4. Pharmacological Intervention

At baseline, participants were randomly assigned to one of three antihypertensive medications (Perindopril, Losartan, or Hydrochlorothiazide (HCTZ)). Perindopril intervention started with a 4-mg/day dose and was titrated to 8 mg/day after 2 weeks. The same scheme was used with Losartan (titration from 50 mg/day to 100 mg/day) and HCTZ (from 12.5 mg/day to 25 mg/day), as previously described [15]. When the starting dose was not tolerated due to issues such as hypotension, cough, or hyperkalemia, participants maintained the lower tolerated dose. Study doses were also adjusted and supervised by a study cardiologist to safely control the blood pressure target (SBP/DBP <140/90 mmHg) [32]. Study medication was loaded into identical capsules to assure double-masking for study staff and participants. Unused study medication was returned for tracking purposes. Where necessary to control blood pressure, the study physician prescribed a supplemental drug (e.g., amlodipine), and the rate of supplemental drug usage was tracked for future study design purposes.

### 2.5. Exercise Intervention

**Figure 1:**
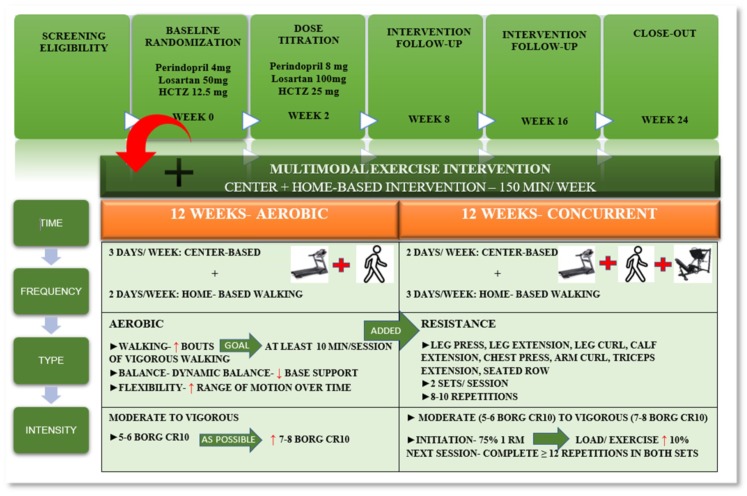
Intervention design characteristics.

In addition to pharmacological intervention, participants also enrolled in a supervised multimodal center and home-based exercise intervention designed to achieve a total of 150 min/week to meet the American College of Sports Medicine guidelines [36]. The intervention was designed with two distinct exercise modes: Aerobic and concurrent [15]. During the aerobic phase (i.e., first 12 weeks), participants engaged in three days/week center-based exercise training and two days/week (30 min/day) of home-based moderate intensity walking. In the concurrent phase (i.e., the last 12 weeks), center-based sessions were reduced to two days/week and home-based walking intervention increased to three days/week. In this phase, a resistance training component was added to investigate potential differences in exercise mode. The intervention was designed to gradually increase volume and intensity while minimizing discomfort and risk of injury. Exercise protocols were designed according to physical activity and exercise guidelines for older adults with hypertension [36,37,38,39] (Figure 1).

In the aerobic phase, exercise sessions began with a brief warm-up followed by 30 min of moderate intensity walking and ended with flexibility and balance exercises. Exercise intensity was monitored with Borg´s category ratio (CR) 10 subjective physical exertion scale [40] and with a heart rate monitor (Polar FT2, Lake Success, NY, USA) according to the guidelines [36]. Walking intensity started on a 5–6 CR 10 scale, and participants were encouraged, as possible, to incorporate brief periods of vigorous walking (7–8 on the CR 10 scale) to target a goal of at least 10 min of vigorous walking/session.

In the concurrent training phase, resistance exercise was added to the center-based intervention. Both lower- and upper-body exercises were performed using standard isotonic resistance equipment (Life Fitness, Schiller Park, IL, USA). Exercises varied by session and included leg press, leg extension, leg curl, calf flexion, chest press, arm curl, triceps extension, and seated row. Resistance training involved two sets of each exercise, with 8–10 repetitions per set. Resistance exercise intensity was designed to progressively increase from moderate (5–6) to vigorous (7–8) throughout the intervention. Intensity started at 75% of participants’ one-repetition maximum (1RM), and the load for a given exercise increased 10% in the next session when the participant was able to complete ≥12 repetitions on both sets.

A home-based walking exercise was also performed at moderate intensity throughout the intervention (5–6 category ratio (CR) 10 scale [40]), and participants self-reported home-based walking in a written log throughout the intervention. In addition, habitual daily physical activity was also objectively measured using a wearable physical activity monitor (SenseWear armband^®^, Body media, Pittsburgh, PA, USA) for 7 days at baseline and at the end of the intervention. Data were sampled in 1-min epochs over 24-h periods to estimate average daily energy expenditure and total minutes in low (<3.0 metabolic equivalents (METS)) and moderate or higher (≥3.0 METS) physical activity, using manufacturer algorithms accordingly with participants’ height, weight, handedness, and smoking status [41,42].

### 2.6. Retention, Adherence, and Safety

Indicators of study feasibility were based on process and scientific purposes [43] and included the integrity of the study protocol, the assessment of treatment safety, and the estimation of treatment effect in primary and secondary outcomes based on the numbers of (a) recruitment and retention rates of the intervention, (b) adherence to the medication and exercise intervention, (c) participants’ safety adherence (adverse events), and (d) estimation of treatment effect on dependent outcomes. These outcomes were chosen to accurately calculate the sample size needed to power the future full RCT [44,45] and help the development and refinement of a manual of procedures.

The success of recruitment and retention rates was measured by the number of participants recruited, the number of withdrawals, and losses to follow-up throughout the intervention. Medication adherence was assessed by study staff and measured through a pill count [46]. Compliance with the exercise intervention was carefully documented by study staff and was measured by the number of sessions attended. Adherence to the home-based intervention was self-reported by participants and was measured by weekly walking minutes with the use of written logs. Lastly, safety was measured by the number and/or seriousness of adverse events attributable to the intervention and was monitored and recorded by study staff during the intervention or spontaneously reported by participants to research staff. Furthermore, to evaluate the safety of the intervention, clinical chemistry and hematology panels were performed at each study visit.

### 2.7. Assessments

Gait speed was assessed from baseline to the 24-week intervention through the 4-m walk test [35], a simple and cost-effective screening tool to assess functional status [6,7,47,48]. Exercise capacity was assessed using the fast-paced 6-minute walk test, a safe and reliable test of exercise capacity in older adults and/or those with cardiovascular conditions [48,49] due to the strong reproducibility and modest correlation with peak VO_2_ [50].

Body composition was assessed using dual X-ray absorptiometry at baseline and at the end of the intervention (24 weeks). Fat mass and fat-free mass were assessed using a fan-bean densitometer (Hologic, Bedford, MA, USA). Body composition analysis was performed in lower and upper body compartments using Lunar Software.

Fasted blood samples were collected at each study visit to assess clinical safety parameters, blood lipids, and a glucose profile. In addition, blood samples were also assayed for prominent biomarkers of inflammation and oxidative stress, including tumor necrosis factor α (TNF-α), interleukin-6 (IL-6), vascular cell adhesion molecule-1 (VCAM-1), endothelium selectin (E-selectin), oxidized low-density lipoprotein (oxLDL), and myeloperoxidase (MPO).

Office blood pressure was manually taken by study staff at each study visit. In addition, throughout the study, participants also recorded home blood pressure twice daily (morning and night) with an automatic digital home blood pressure monitor (Microlife, Dunedin, FL, USA) that met independent validity standards [51].

### 2.8. Statistical Analyses

Analyses were performed in R version 3.5.1 following a published protocol [15]. As recommended by the Consolidated Standards of Reporting Trials (CONSORT), we reported estimates of change over time with 95% confidence intervals (CIs) and did not conduct formal hypothesis testing, as previously outlined [44,45]. Participant characteristics were computed overall and by treatment group. Data from all randomized participants were analyzed, regardless of adherence (i.e., the intent-to-treat principle). Linear mixed models were applied to estimate change over time, adjusted for age, sex, and baseline outcome measure, in primary and secondary outcomes overall and by treatment group [52,53]. Normality of error terms was assessed using the Shapiro-Wilks test. Log-transformation was applied to outcomes as needed to achieve normally distributed error distributions.

## 3. Results

### 3.1. Participant Recruitment and Randomization

**Figure 2:**
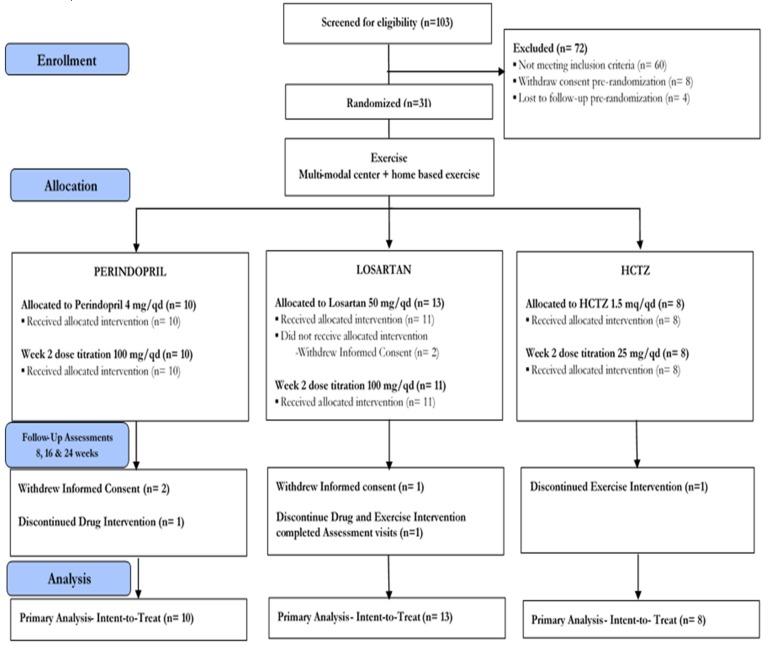
Participant flow through the study.

The screening process began in May 2014 and ended in August 2016. The first participant was randomized on 5 May 2014 and the last on 9 August 2016. The trial ended in July 2017 due to the end of financial support. A total of 103 participants were screened for eligibility (Figure 2). Of these, 31 participants (30%) met eligibility screening criteria, provided written informed consent, and completed baseline assessment procedures. Sixty participants (58%) were excluded based on screening study entry criteria. Eight participants (8%) withdrew informed consent prior to randomization, and 4 participants (6%) were lost to follow-up pre-randomization. Thirty-one participants were randomly assigned to the intervention (Perindopril = 10; Losartan = 13; HCTZ = 8).

### 3.2. Retention

After randomization, five (16%) participants withdrew informed consent (Perindopril = 2; Losartan = 3; Figure 2). One participant discontinued pharmacological intervention (Perindopril = 1), and one discontinued exercise intervention (HCTZ = 1). Moreover, one participant from the Losartan group discontinued both the drug and exercise interventions but completed study assessment visits. Overall, 26 (retention rate: 84%) participants completed the study (Perindopril = 8; Losartan = 11; HCTZ = 8).

### 3.3. Adherence and Safety

Overall, adherence to pharmacological intervention was 93% (Perindopril = 90%; Losartan = 94%; HCTZ = 97%), and 19% of participants (Perindopril = 1; Losartan = 3; HCTZ = 2) used a second antihypertensive medication to control blood pressure (Amlodipine = 3 (5 mg = 1; 10 mg = 2); Metoprolol 25 mg = 2; Nifedipine 90 mg = 1). Furthermore, the overall compliance with the center-based exercise intervention was 83% (Perindopril = 87%; Losartan = 79%; HCTZ = 84%) and with home-based exercise was 91.4 (62.6, 120.2) min/week (Perindopril: 92.0 (55.1, 128.9); Losartan: 98.4 (21.7, 175.1); HCTZ: 82.6 (24.0, 141.1)). Furthermore, groups exhibited similar average range changes over time in daily energy expenditure and in physical activity patterns. The average change in daily energy expenditure ranged from −184 (Losartan 95% CI: −337, −30) cal/day to 24 (HCTZ: −136, 184) cal/day. Further, the average change in low physical activity ranged from −13.2 (Losartan: −32.3, 5.8) min/day to −1.2 (HCTZ: −22.2, 19.8) min/day and in moderate or higher physical activity ranged from −22.9 (Losartan: −37.6, −8.3) min/day to −4.4 (HCTZ: −20.4, 11.6) min/day.

Regarding safety, only one serious adverse event was observed and was unrelated to the study (one participant required hospital admission due to rectal bleeding). Following randomization, 29 adverse events related or possibly related to the intervention were reported (Perindopril = 5; Losartan = 12; HCTZ = 11). Observed adverse events were categorized as expected based upon the study interventions, with the most frequent being musculoskeletal issues (*n* = 10), spontaneous reports of elevated blood pressure (*n* = 7), swelling (*n* = 3), falls (*n* = 2), and cough (*n* = 2). Moreover, groups presented similar average range changes in comprehensive blood chemistry and in complete blood count outcomes, and values were within the reference range [54] (Supplementary Materials, Table S2).

### 3.4. Participant Characteristics

Baseline characteristics are shown in Table 1. At baseline, 68% of the sample was Caucasian and 61% were female. Overall, the study sample had a mean age (standard deviation (SD)) of 70.6 (6.1) years and used an average of 3.7 (1.6) medications. As required by the protocol, the eligible participants were functionally impaired (4-m walk test: 0.97 (0.15) m/s) and physically inactive (CHAMPS: 51.0 (51.1) min/week) and were not cognitively impaired (Mini Mental State Examination: 27.8 (1.5) points).

### 3.5. Gait Speed

**Figure 3:**
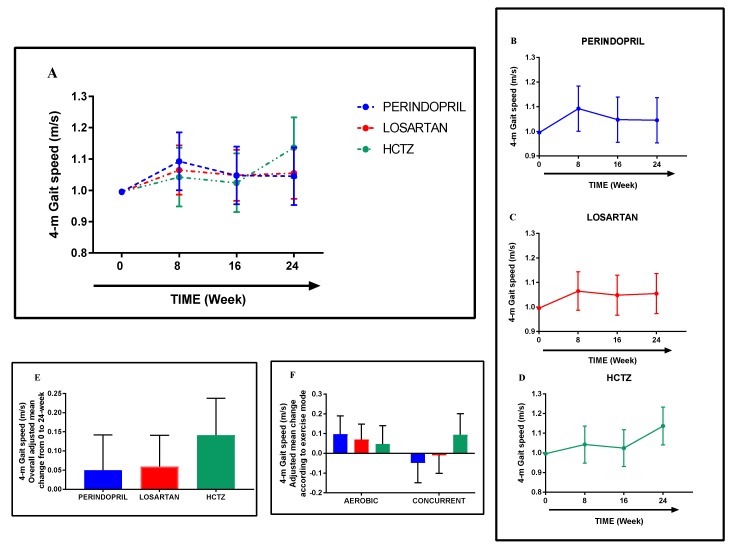
Adjusted mean change in gait speed from baseline to 24-week follow-up according to the randomization group (**A**–**D**). Overall adjusted mean change was within groups (**E**) and according to exercise mode (**F**). Data are expressed as adjusted mean change within groups with 95% confidence intervals adjusted to age, sex, and baseline status.

From pre-intervention (week 0) to post-intervention (week 24), the estimated overall change in gait speed was 0.06 (95% CI: −0.01, 0.13) m/s, with adjusted mean changes within groups ranging from 0.05 (Perindopril: −0.04, 0.14) m/s to 0.14 (HCTZ: 0.05, 0.24) m/s (Figure 3A–E). Interestingly, though as expected not statistically significant, directional changes within groups may have been influenced by exercise mode as changes in the aerobic phase ranged from 0.05 (HCTZ: −0.05, 0.14) m/s to 0.10 (Perindopril: 0.01, 0.19) m/s and in the concurrent phase ranged from −0.05 (Perindopril: −0.15, 0.05) m/s to 0.09 (HCTZ: −0.01, 0.20) m/s (Figure 3F).

### 3.6. Blood Pressure, Body Composition, and Exercise Capacity

**Figure 4:**
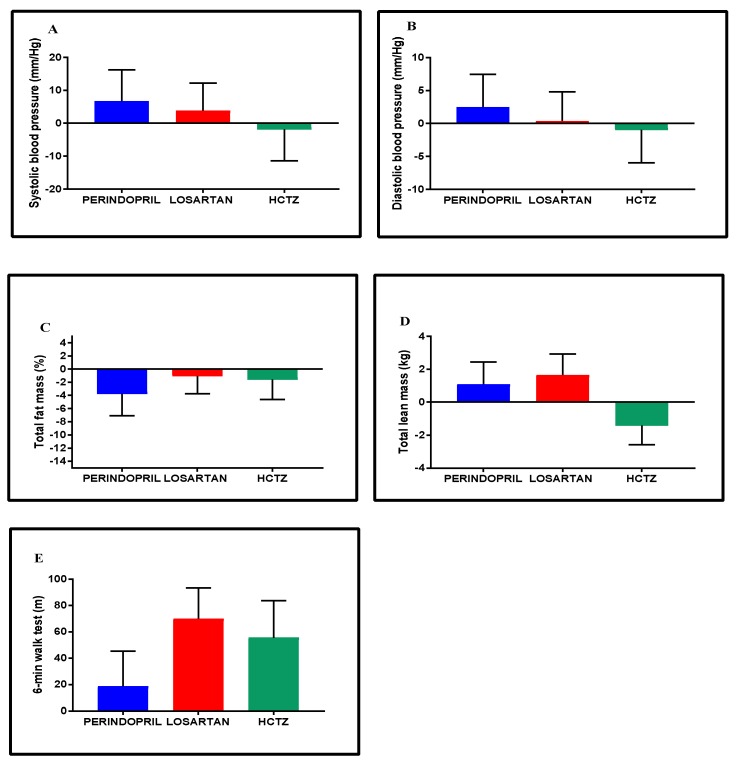
Adjusted mean change within groups in blood pressure (**A**,**B**), body composition (**C**,**D**), and exercise capacity (**E**) from baseline to 24-week follow-up. Data are expressed as adjusted mean change within groups with 95% confidence intervals adjusted to age, sex, and baseline status.

Throughout the intervention, the target range of blood pressure was achieved across groups. Overall, groups exhibited similar average range changes in blood pressure, body composition, and exercise capacity (Figure 4A–E). Though not significant, potential differences in the patterns of change among groups may have existed, particularly in HCTZ. For instance, the average change in SBP, in DBP, and in total lean mass in HCTZ (−1.8 (−11.4, 7.8) mmHg; −1.0 (−6.0, 4.0) mmHg; and −1.4 (−2.6, −0.3) kg, respectively) were directionally contrary to the changes in the Perindopril and Losartan groups.

### 3.7. Clinical Metabolic Profiles

**Figure 5:**
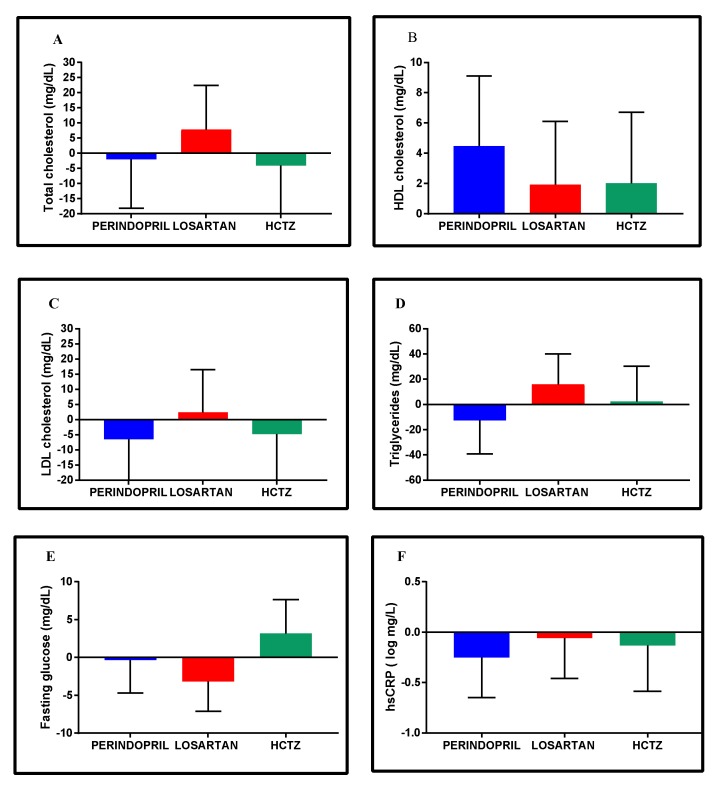
Adjusted mean change within groups in lipid (**A–D**) and fasting glucose profiles (**E**) and in high-sensitivity C-reactive protein (**F**) from baseline to 24-week follow-up. Data are expressed as adjusted mean change within groups with 95% confidence intervals adjusted to age, sex, and baseline status. HDL cholesterol: High-density lipoprotein cholesterol; hsCRP: High-sensitivity C-reactive protein; LDL cholesterol: Low-density lipoprotein cholesterol. Log-transformation was used to normalize data distribution (Shapiro–Wilks test, *p* < 0.05).

Overall, changes in metabolic profiles were generally similar across groups, with a few possible exceptions where directional differences were observed (Figure 5A–F). For instance, the average change in total cholesterol and in low-density lipoprotein (LDL) cholesterol in the Losartan group was respectively +7.9 (−6.7, 22.4) mg/day and +2.4 (−11.6, 16.5) mg/dL, compared to directional declines in the other groups.

### 3.8. Inflammatory and Oxidative Stress Biomarkers

**Figure 6:**
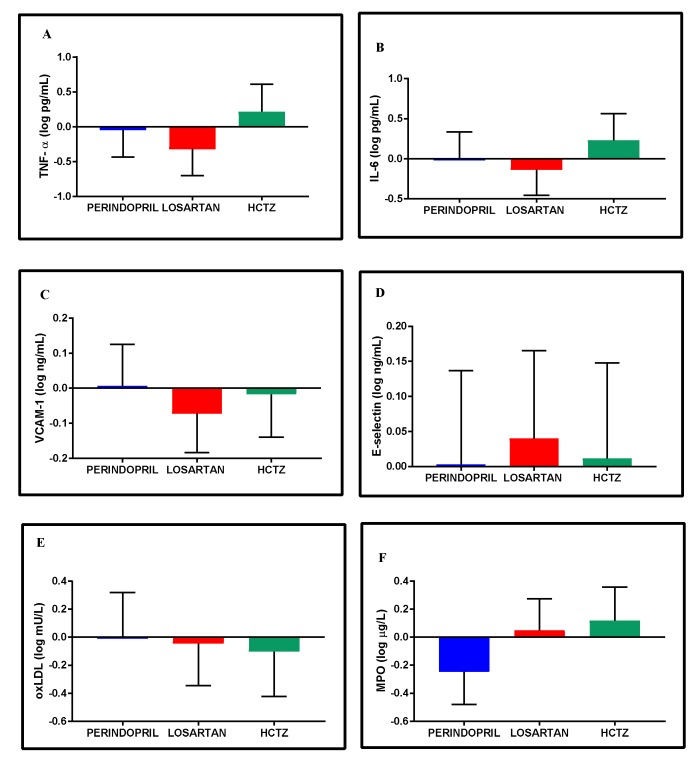
Adjusted mean change within groups in inflammatory (**A–D**) and oxidative stress biomarkers (**E**,**F**) from baseline to 24-week follow-up. Data are expressed as adjusted mean change within groups with 95% confidence intervals adjusted to age, sex, and baseline status. Log-transformation was used to normalize data distribution (Shapiro-Wilks test, *p* < 0.05). IL-6: Interleukin-6; MPO: Myeloperoxidase; oxLDL: Oxidized LDL; TNF-α: Tumor necrosis factor-α; VCAM-1: Vascular cell adhesion molecule-1.

Throughout the intervention, the overall average range changes in the inflammatory and oxidative stress biomarkers were modest and generally similar across groups (Figure 6A–F). Notable results included the mean change in the HCTZ group in the inflammatory biomarkers TNF-α (+0.2 (−0.2, 0.6) log pg/mL) and IL-6 (+0.2 (−0.1, 0.6) log pg/mL) and the mean change in MPO observed in the Perindopril group (−0.3 ( −0.5, −0.0) log μg/L).

### 3.9. Exercise Mode

Similarly to gait speed, we explored the potential impact of exercise mode on changes in secondary study outcomes, with some interesting findings observed (Table 2). For instance, in exercise capacity, the average changes in the Perindopril group ranged from +45.8 (19.1, 72.6) m in the aerobic phase to −27.1 (−55.9, 1.6) m during the concurrent phase, while the Losartan group ranged from +67.5 (aerobic: 44.7, 90.3) m to +2.3 (concurrent: −23.2, 27.8) m. Additional changes in study outcomes, separated by study phase (i.e., exercise mode), are shown in Table 2.

## 4. Discussion

This three-arm, triple-masked pilot RCT assessed feasibility, safety, and protocol integrity to support the conduct of a fully powered RCT to evaluate the efficacy of different antihypertensive medications to improve functional status in hypertensive seniors, when combined with exercise [15]. Our preliminary data indicated satisfactory recruitment and retention rates and good adherence rates to the pharmacological and exercise intervention, and there was no evidence of increased risk for adverse events in any treatment group relative to the others. Moreover, our results also suggested potential evidence of different patterns of change over time with respect to treatment groups and exercise mode, particularly in gait speed. Thus, this 24-week multimodal intervention designed to improve physical function in functionally impaired older adults is feasible and safe, and can be implemented as a full-scale RCT.

One of the most important aspects of the trial was to determine the safety and feasibility of the protocol in the target population (functionally impaired hypertensive older adults). As expected in this study population [55], a number of adverse events were reported, many of which were anticipated for this high-risk population (e.g., elevated blood pressure (*n* = 7), swelling (*n* = 3), and cough (*n* = 2)). However, only one serious adverse event was recorded and was determined to be unrelated to the study intervention. These data support the application of the pilot RCT’s protocol in a full-scale RCT. Moreover, the trial demonstrated a good overall adherence to both the pharmacologic and exercise interventions (>90% and >79%, respectively). Furthermore, the retention rate (84% of the participants randomized) was satisfactory and provides an overall estimate for projecting drop-out in a full-scale RCT and for accurately calculating the sample size needed to power the future RCT [31,56,57]. Additionally, the overall mean change in gait speed across groups of 0.06 m/s (−0.01, 0.13) was a positive indicator of intervention feasibility and efficacy, demonstrating the overall benefit of the study intervention given that increases >0.05 m/s have been associated with meaningful improvements in the performance of the activities of daily life and a decreased risk of functional impairment in advanced age [58,59].

One of the challenges experienced in the trial was participant recruitment, commonly one of the most challenging aspects of study execution [60,61]. The trial was conducted in a relatively small metropolitan area that, coupled with a relatively significant time commitment (24 weeks, two to three days/week), may have limited overall recruitment in the trial. Potential solutions may include multiple sites for recruitment and/or implementing the study in a larger metropolitan area. Moreover, the study provided important information regarding the substantial financial and time resources necessary to randomize each participant, indicating that a fully powered trial will require significant financial resources and a longer time period to successfully execute such a trial. Another common challenging barrier to study recruitment in trials is study entry criteria [60]. In this pilot, approximately one-third of the screened participants fulfilled all of our study entry criteria, an indicator of study feasibility when compared to previous pilot studies [62]. Interestingly, the majority of the exclusions at the screening visit were based on a lack of an objective functional physical limitation (18%), regular physical activity (15%), or the presence of a medical condition that precluded safety enrollment in the intervention (10%). Given that the objective was to improve function via structured physical activity (i.e., exercise), it is important to keep these criteria to prevent potential ceiling effects.

Notably, one important change occurred during the study period that should improve recruitment in future studies. During the study, clinical recommendations changed related to antihypertensive prescription for persons with type 2 diabetes (T2D) [63,64], broadening the available first-line therapies for those with T2D. This group represented a significant portion of those excluded from the study, and thus this change should enhance recruitment in the future trial. Moreover, we excluded persons with known hypersensitivity to ACE inhibitors from this trial. It is possible that a future trial might include separate randomization strata wherein these individuals are randomized to one of the other two drugs, to facilitate broader participation in the study.

As previously outlined [15], the use of supplemental medication to control blood pressure in participants unable to reach target ranges of blood pressure was tracked and used to refine the design of the future trial. Throughout the intervention, 19% of participants required a supplemental drug prescription. During the pilot, supplemental drug prescription was left to the study physician’s discretion, and thus it varied between individuals. However, the future trial could be enhanced by standardizing the supplemental drug choice and dosages and integrating them tightly with the first-line medication provided.

Regarding exercise intervention, two different modes were used to evaluate potential differences in outcomes, per prior evidence in this area [31,65]. Though not statistically significant per the pilot study design, directional changes in outcomes suggested that, as prior evidence has suggested, exercise mode may influence study outcomes. Notably, the average change in the 4-m gait speed in the Perindopril group ranged from +0.10 m/s in the aerobic phase to −0.05 m/s during the concurrent phase, with similar evidence observed for exercise capacity, blood pressure, and systemic cardiometabolic biomarkers. Collectively, these preliminary data seem to suggest that the aerobic exercise mode may be more appropriate to test our central hypothesis, which is consistent with previous preclinical studies [27]. Thus, future studies may need to isolate exercise modalities to specifically answer questions related to efficacy differences in combination with specific antihypertensive medications, particularly studies using ACE inhibitors and angiotensin receptor blockers. Moreover, the home-based exercise intervention in the study was assessed via self-report. Future RCTs are likely to benefit from the use of objective devices to measure home physical activity to decrease social desirability and nonresponse bias [61]. Furthermore, the use of objective measures would also prevent participant burden and would accurately calculate adherence to home physical activity since self-reported methods tend to overestimate physical activity levels compared to objective measurement devices [66,67].

Interestingly, after the intervention, groups exhibited potential different directional patterns in the lipid profile. Previous preclinical studies [68,69] have suggested an interaction between lipid metabolism and the renin-angiotensin system [69] and have suggested that both ACE inhibitors and angiotensin receptor antagonists (e.g., Losartan) have a beneficial lipid lowering effect [69,70,71,72] through the inhibition of LDL oxidation and macrophage cholesterol biosynthesis [68,69]. Although our results supported directional declines in the Perindopril group, our pilot data did not suggest changes in total cholesterol and LDL cholesterol in the Losartan group. These contradictory findings may have been due to differences in study intervention length (4-week short-term [70] vs. our 12-week long-term intervention), participant baseline clinical characteristics (diabetic nephropathic patients and/or with dyslipidemia [70,71] vs. our participants in a normal range), and the drug used (Telmisartan [72] vs. Losartan). Furthermore, differences in the underlying mechanism(s) of these different antihypertensive medications may have contributed to the potential directional differences found between the Perindopril and Losartan groups in our pilot study. Although speculative, prior preclinical evidence [69,73] has suggested that angiotensin receptor antagonists may improve dyslipidemia by reducing triglycerides through the activation of peroxisome proliferator-activated receptor (PPAR) gamma, which regulates lipid metabolism [69] and influences the expression of PPARgamma target genes involved in carbohydrate and lipid metabolism reducing glucose, insulin, and triglyceride levels [73]. Remarkably, Benson and colleagues [73] have reported that apart from Telmisartan, which has a structurally unique characteristic that allowed it to function as a partial agonist of PPARgamma, none of the other angiotensin receptor antagonists appeared to activate PPARgamma when tested at concentrations typically achieved in plasma with conventional oral dosing. Notably, triglyceride results in the Losartan group (+16.1 (−7.8, 40.0) mg/dL) seem to support this rationale and may explain the directional changes in the lipid profile in the Losartan group in our trial. Hence, these results suggest that ACE inhibitors and angiotensin receptor antagonists may have different underlying mechanism(s) in lipid metabolism and that the Losartan mechanism (or mechanisms) is not well understood.

Lipid metabolism is associated with atherosclerotic plaque [74], which is associated with increases in blood pressure [63], the accumulation of inter- and intramuscular adipose tissue, and the decline of skeletal muscle quality and function [8,75]. Thus, understanding how antihypertensive therapies influence these outcomes in conjunction with exercise could provide further mechanistic insight into potential changes in physical function. Thus, lipid metabolism may represent an important target for a future trial in this area.

In summary, this study indicated that a study protocol randomizing first-line antihypertensive medications combined with exercise training among functionally impaired hypertensive older adults is safe and feasible, although several challenges (particularly in the area of recruitment) must be considered and overcome in the execution of a larger-scale trial. As a pilot, data from the study should not be overinterpreted, but they do provide important preliminary data necessary for the design of a fully powered RCT. Several important considerations were learned from this trial, including those related to study entry criteria, participant recruitment, randomization, and intervention implementation, and they should benefit the design of a future trial in this area. Such a trial may ultimately address, at least for specific exercise modes, long-standing questions regarding the efficacy of exercise among older persons when combined with differing antihypertensive medications.

## Figures and Tables

**Table 1:** Baseline characteristics of the study sample according to the randomization group.

Outcomes	Total (*n* = 31)	Perindopril (*n* = 10)	Losartan (*n* = 13)	HCTZ (*n* = 8)
Female, %	61.3	50.0	69.2	62.5
Age, years	70.6 (6.1)	72.9 (7.2)	71.0 (6.2)	67.1 (2.2)
MMSE	27.8 (1.5)	27.9 (1.3)	27.8 (1.7)	27.6 (1.5)
Race, Caucasian, %	67.7	80.0	69.2	50.0
Education, college graduate, %	54.8	60.0	53.8	50.0
Measurements				
Weight, kg	91.3 (14.3)	88.6 (14.8)	93.6 (16.4)	91.0 (10.9)
Body mass index, kg/m^2^	33.4 (6.0)	31.7 (6.6)	34.5 (6.4)	33.8 (4.5)
Total body fat mass, kg	38.5 (11.4)	37.1(12.2)	40.3 (12.6)	37.6 (9.2)
Total lean mass, kg	48.0 (7.4)	47.2 (7.7)	48.5 (8.8)	48.1 (5.2)
Systolic blood pressure, mmHg	133.8 (15.3)	133.2 (10.9)	134.8 (14.5)	132.9 (22.2)
Diastolic blood pressure, mmHg	81.1 (9.1)	79.3 (4.6)	81.2 (10.0)	83.1 (12.3)
Total cholesterol, mg/dL	187.1 (37.3)	187.6 (29.6)	193.2 (43.9)	176.4 (36.3)
HDL cholesterol, mg/dL	58.4 (14.0)	63.2 (10.4)	56.2 (15.6)	55.9 (15.5)
LDL cholesterol, mg/dL	105.0 (31.3)	100.1 (27.5)	111.5 (37.6)	100.6 (26.0)
Triglycerides, mg/dL	122.1 (50.5)	121.5 (49.6)	134.9 (58.4)	101.9 (33.7)
Fasting glucose, mg/dL	95.7 (9.5)	93.9 (6.7)	96.4 (12.1)	96.8 (8.4)
Creatinine, mg/dL	0.9 (0.2)	0.9 (0.1)	1.0 (0.2)	0.9 (0.2)
BUN/Creatinine ratio, mg/dL	20.2 (6.3)	20.7 (4.5)	19.1 (6.9)	21.2 (7.5)
Functional Measures				
6-min walk distance, m	394.8 (80.9)	392.5 (72.2)	388.1 (97.8)	408.8 (68.2)
SPPB	10.5 (1.3)	10.1 (1.5)	10.4 (1.4)	11.1 (0.8)
CHAMPS, min/week	51.0 (51.1)	46.0 (48.4)	50.8 (46.2)	57.5 (66.5)
4-m gait speed, m/s	0.97 (0.15)	0.95 (0.17)	0.98 (0.14)	0.99 (0.13)
Energy Expenditure				
Energy expenditure, cal/day	2267 (352)	2279 (375)	2492 (647)	2162 (269)
Low PA, min/day	283.0 (190.1)	317.6 (171.8)	442.8 (226.4)	236.2 (137.4)
Moderate or higher PA, min/day	46.9 (30.0)	55.0 (30.5)	72.8 (30.2)	41.2 (22.7)
Dietary intake				
Total intake, kcal/day	1737 (569)	1938 (508)	1624 (725)	1655 (304)
Protein, gr/day	68.9 (21.4)	73.9 (17.7)	63.6 (26.7)	70.8 (17.2)
Carbohydrate, gr/day	215.9 (67.8)	240.7 (64.6)	206.3 (77.4)	199.0 (54.5)
Sugar, gr/day	77.8 (30.0)	78.1 (25.5)	79.5 (35.9)	74.6 (29.4)
Fat, gr/day	69.2 (33.4)	74.9 (31.3)	62.5 (42.9)	72.2 (17.7)
Vitamin D, μg	2.76 (4.74)	2.79 (3.14)	3.61 (6.90)	1.37 (1.18)
Medication				
Total medication, *n*	3.7 (1.6)	3.8 (1.8)	3.9 (1.7)	3.4 (1.5)
Antidyslipidemic medication, %	45.2	50.0	30.8	62.5
ACEs, %	32.3	50.0	30.8	12.5
ARA, %	22.6	10.0	23.1	37.5

Notes: ACEs: Angiotensin-converting enzyme inhibitors. ARA: Angiotensin receptor antagonist. BUN: Blood urea nitrogen. CHAMPS: Community Health Activities Model Program for Seniors questionnaire. HDL: High-density lipoprotein. LDL: Low-density lipoprotein. MMSE: Mini Mental State Examination. PA: Physical activity. SPPB: Short physical performance battery. HCTZ: Hydrochlorothiazide. Physical activity was defined as low (<3.0 metabolic equivalents (METS)) and as moderate or higher (≥3.0 METS). Data are expressed as mean (SD), count, or percentage as appropriate.

**Table 2:** Change within groups in secondary outcomes according to exercise mode intervention.

Outcomes	Perindopril		Losartan		HCTZ	
	Aerobic	Concurrent	Aerobic	Concurrent	Aerobic	Concurrent
	Baseline-8 Weeks	8–24 Weeks	Baseline-8 Weeks	8–24 Weeks	Baseline-8 Weeks	8–24 Weeks
6-min walk distance, m	45.8 (19.1, 72.6)	−27.1 (−55.9, 1.6)	67.5 (44.7, 90.3)	2.3 (−23.2, 27.8)	40.0 (8.9, 63.2)	19.6 (−10.6, 49.7)
SBP, mmHg	5.0 (−4.4, 14.5)	1.8 (−11.7, 15.2)	8.9 (0.8, 17.0)	−5.1 (−16.9, 6.7)	−2.3 (−11.9, 7.3)	0.5 (−12.9, 13.9)
DBP, mmHg	1.1 (−3.8, 6.1)	1.4 (−7.7, 10.4)	3.4 (−0.8, 7.6)	3.1 (−11.0, 4.9)	2.4 (−2.6, 7.4)	3.4 (−12.4, 5.7)
Total cholesterol, mg/dL	−11.7 (−27,9, 4.6)	9.8 (−11.5, 40.0)	−8.3 (−22.3, 5.6)	16.2 (−2.5, 34.9)	−7.3 (−24.6, 10.1)	3.3 (−18.9, 25.5)
HDL cholesterol, mg/dL	−0.3 (−4.9, 4.4)	4.8 (−0.4, 9.9)	2.1 (−1.9, 6.2)	−0.2 (−4.8, 4.4)	−0.3 (−5.1, 4.6)	2.3 (−3.2, 7.7)
LDL cholesterol, mg/dL	−9.8 (−25.7, 6.0)	3.4 (−16.4, 23.2)	−9.1 (−22.5, 4.5)	11.5 (−6.0, 28.9)	−7.4 (−24.1, 9.3)	2.6 (−18.1, 23.4)
Triglycerides, mg/dL	−22.0 (−48.8, 4.8)	9.5 (−25.2, 44.2)	−6.7 (−29.6, 16.2)	22.8 (−7.7, 53.3)	3.7 (−25.7, 33.1)	−1.3 (−37.6, 35.0)
Fasting glucose, mg/dL	1.0 (−3.5, 5.4)	−1.3 (−8.4, 5.9)	−3.6 (−7.3, 0.2)	0.4 (−5.8, 6.7)	−1.2 (−5.6, 3.3)	4.4 (−2.7, 11.5)
hsCRP, log mg/L	0.2 (−0.2, 0.7)	−0.5 (−1.1, 0.2)	−0.1 (−0.5, 0.3)	0.1(−0.5, 0.6)	−0.1 (−0.6, 0.3)	0.0 (−0.7, 0.7)
TNF-α, log pg/mL	0.2 (−0.2, 0.6)	−0.3 (−0.8, 0.2)	−0.0 (−0.4, 0.3)	−0.3 (−0.8, 0.2)	0.1 (−0.3, 0.5)	0.1 (−0.4, 0.6)
IL-6, log pg/mL	0.0 (−0.3, 0.4)	−0.0 (−0.6, 0.6)	−0.0 (−0.3, 0.3)	−0.1 (−0.6, 0.4)	0.3 (0.0, 0.7)	−0.1 (−0.7. 0.4)
VCAM-1, log ng/mL	0.0 (−0.1, 0.2)	−0.0 (−0.2, 0.1)	−0.1 (−0.2, −0.0)	0.0 (−0.1, 0.2)	−0.0 (−0.2, 0.1)	0.0 (−0.2, 0.2)
E-selectin, log ng/mL	0.1 (−0.1, 0.2)	−0.1 (−0.2, 0.1)	0.0 (−0.1, 0.1)	0.0 (−0.1, 0.2)	−0.1 (−0.3, 0.0)	0.1 (−0.0, 0.3)
oxLDL, log mU/L	−0.3 (−0.6, 0.0)	0.3 (−0.2, 0.8)	−0.2 (−0.5, 0.1)	0.2 (−0.3, 0.6)	−0.1 (−0.4, 0.2)	0.0 (−0.5, 0.5)
MPO, log μg/L	−0.1 (−0.3, 0.2)	−0.2 (−0.4, 0.1)	−0.1 (−0.4, 0.1)	0.2 (−0.1, 0.4)	−0.1 (−0.3, 0.2)	0.2 (−0.1, 0.5)

Notes: DBP: Diastolic blood pressure; HDL: High-density lipoprotein; hsCRP: High-sensitivity C- reactive protein; IL-6: Interleukin- 6; LDL: Low-density lipoprotein; MPO: Myeloperoxidase; oxLDL: Oxidized LDL; SBP: Systolic blood pressure; SPPB: Short physical performance battery; TNF-α: Tumor necrosis factor- α; VCAM-1: Vascular cell adhesion molecule-1. Data are expressed as within-groups adjusted mean change with 95% confidence intervals, adjusted to age, sex, and baseline status. Log-transformation was used to normalize data distribution (Shapiro-Wilks test, *p* < 0.05).

## Supplementary Materials

The following are available online at http://www.mdpi.com/2077-0383/8/2/196/s1, Table S1: Additional exclusion criteria [76,77], Table S2: Change in clinical safety parameters from baseline to 24-week follow-up according to the randomization group.

## References

[1] Muszalik M., Dijkstra A., Kędziora-Kornatowska K., Zielińska-Więczkowska H., Kornatowski T. (2011). Independence of elderly patients with arterial hypertension in fulfilling their needs, in the aspect of functional assessment and quality of life (QoL). Arch. Gerontol. Geriatr..

[2] Newman A.B., Simonsick E.M., Naydeck B.L., Boudreau R.M., Kritchevsky S.B., Nevitt M.C., Pahor M., Satterfield S., Brach J.S., Studenski S.A. (2006). Association of long-distance corridor walk performance with mortality, cardiovascular disease, mobility limitation, and disability. JAMA.

[3] Shaw L.J., Olson M.B., Kip K., Kelsey S.F., Johnson B.D., Mark D.B., Reis S.E., Mankad S., Rogers W.J., Pohost G.M. (2006). The value of estimated functional capacity in estimating outcome: Results from the NHBLI-Sponsored Women’s Ischemia Syndrome Evaluation (WISE) Study. J. Am. Coll. Cardiol..

[4] Studenski S., Perera S., Patel K. (2011). Gait Speed and Survival in Older Adults. JAMA.

[5] McGinn A.P., Kaplan R.C., Verghese J., Rosenbaum D.M., Psaty B.M., Baird A.E., Lynch J.K., Wolf P.A., Kooperberg C., Larson J.C. (2008). Walking speed and risk of incident ischemic stroke among postmenopausal women. Stroke.

[6] Afilalo J., Eisenberg M.J., Morin J.-F., Bergman H., Monette J., Noiseux N., Perrault L.P., Alexander K.P., Langlois Y., Dendukuri N. (2010). Gait speed as an incremental predictor of mortality and major morbidity in elderly patients undergoing cardiac surgery. J. Am. Coll. Cardiol..

[7] Dumurgier J., Elbaz A., Ducimetiere P., Tavernier B., Alperovitch A., Tzourio C. (2009). Slow walking speed and cardiovascular death in well functioning older adults: Prospective cohort study. BMJ.

[8] Cruz-Jentoft A.J., Bahat G., Bauer J., Boirie Y., Bruyere O., Cederholm T., Cooper C., Landi F., Rolland Y., Sayer A.A. (2018). Sarcopenia: Revised European consensus on definition and diagnosis. Age Ageing.

[9] Peel N.M., Kuys S.S., Klein K. (2013). Gait speed as a measure in geriatric assessment in clinical settings: A systematic review. J. Gerontol.-Ser. A Biol. Sci. Med. Sci..

[10] Rosano C., Longstreth W.R.B., Taylor C.A., Du Y., Kuller L.H., Newman A.B. (2011). High Blood Pressure Accelerates Gait Slowing in Well- Functioning Older Adults over 18-Years of Follow-Up. J. Am. Geriatr. Soc..

[11] Balzi D., Lauretani F., Barchielli A., Ferrucci L., Bandinelli S., Buiatti E., Milaneschi Y., Guralnik J.M. (2009). Risk factors for disability in older persons over 3-year follow-up. Age Ageing.

[12] Dumurgier J., Elbaz A., Dufouil C., Tavernier B., Tzourio C. (2010). Hypertension and lower walking speed in the elderly: The Three-City study. J. Hypertens..

[13] Buford T.W., Manini T.M., Hsu F.-C., Cesari M., Anton S.D., Nayfield S., Stafford R.S., Church T.S., Pahor M., Carter C.S. (2012). Angiotensin-converting enzyme inhibitor use by older adults is associated with greater functional responses to exercise. J. Am. Geriatr. Soc..

[14] Hajjar I., Lackland D.T., Cupples L.A., Lipsitz L.A. (2007). Association between concurrent and remote blood pressure and disability in older adults. Hypertension.

[15] Buford T.W., Anton S.D., Bavry A.A., Carter C.S., Daniels M.J., Pahor M. (2015). Multi-modal intervention to reduce cardiovascular risk among hypertensive older adults: Design of a randomized clinical trial. Contemp. Clin. Trials.

[16] United Nations Department of Economic and Social Affairs/Population Division (2015). World Population Ageing.

[17] Mendis S., Puska P., Norrving B. (2011). Global Atlas on Cardiovascular Disease Prevention and Control.

[18] Buford T.W. (2016). Hypertension and aging. Ageing Res. Rev..

[19] Hodgson T.A., Cohen A.J. (1999). Medical care expenditures for selected circulatory diseases: Opportunities for reducing national health expenditures. Med. Care.

[20] Simon C.B., Lee-McMullen B., Phelan D., Gilkes J., Carter C.S., Buford T.W. (2015). The renin-angiotensin system and prevention of age-related functional decline: Where are we now?. Age (Dordr).

[21] McAlister F.A. (2012). Angiotensin-converting enzyme inhibitors or angiotensin receptor blockers are beneficial in normotensive atherosclerotic patients: A collaborative meta-analysis of randomized trials. Eur. Heart J..

[22] Cranney A. (2007). Is there a new role for angiotensin-converting-enzyme inhibitors in elderly patients?. CMAJ.

[23] Sumukadas D., Struthers A.D., McMurdo M.E.T. (2006). Sarcopenia—A potential target for Angiotensin-converting enzyme inhibition?. Gerontology.

[24] Carter C.S., Onder G., Kritchevsky S.B., Pahor M. (2005). Angiotensin-converting enzyme inhibition intervention in elderly persons: Effects on body composition and physical performance. J. Gerontol. A Biol. Sci. Med. Sci..

[25] Sica D. (2011). Are There Pleiotropic Effects of Antihypertensive Medications or Is It All About the Blood Pressure in the Patient with Diabetes and Hypertension?. J. Clin. Hypertens..

[26] Yusuf S., Sleight P., Pogue J., Bosch J., Davies R., Dagenais G. (2000). Effects of an angiotensin converting- enzyme inhibitor, ramipril, on cardiovascular events in high-risk patients. N. Engl. J. Med..

[27] Carter C.S., Marzetti E., Leeuwenburgh C., Manini T., Foster T.C., Groban L., Scarpace P.J., Morgan D. (2012). Usefulness of preclinical models for assessing the efficacy of late-life interventions for sarcopenia. J. Gerontol.-Ser. A Biol. Sci. Med. Sci..

[28] Morley J.E., von Haehling S., Anker S.D. (2014). Are we closer to having drugs to treat muscle wasting disease?. J. Cachexia Sarcopenia Muscle.

[29] Morley J.E. (2016). Pharmacologic Options for the Treatment of Sarcopenia. Calcif. Tissue Int..

[30] Spira D., Walston J., Buchmann N., Nikolov J., Demuth I., Steinhagen-Thiessen E., Eckardt R., Norman K. (2016). Angiotensin-Converting Enzyme Inhibitors and Parameters of Sarcopenia: Relation to Muscle Mass, Strength and Function: Data from the Berlin Aging Study-II (BASE-II). Drugs Aging.

[31] Sumukadas D., Band M., Miller S., Cvoro V., Witham M., Struthers A., McConnachie A., Lloyd S.M., McMurdo M. (2014). Do ACE inhibitors improve the response to exercise training in functionally impaired older adults? A randomized controlled trial. J. Gerontol. A. Biol. Sci. Med. Sci..

[32] Chobanian A.V., Bakris G.L., Black H.R., Cushman W.C., Green L.A., Izzo J.L.J., Jones D.W., Materson B.J., Oparil S., Wright J.T.J. (2003). The Seventh Report of the Joint National Committee on Prevention, Detection, Evaluation, and Treatment of High Blood Pressure: The JNC 7 report. JAMA.

[33] Tremblay M.S., Aubert S., Barnes J.D., Saunders T.J., Carson V., Latimer-Cheung A.E., Chastin S.F.M., Altenburg T.M., Chinapaw M.J.M. (2017). Sedentary behavior research network (SBRN)–Terminology consensus project process and outcome. Int. J. Behav. Nutr. Phys. Act..

[34] Stewart A.L., Mills K.M., King A.C., Haskell W.L., Gillis D., Ritter P.L. (2001). CHAMPS Physical Activity Questionnaire for Older Adults: Outcomes for interventions. Med. Sci. Sport. Exerc..

[35] Rolland Y.M., Cesari M., Miller M.E., Penninx B.W., Atkinson H.H., Pahor M. (2004). Reliability of the 400-M Usual-Pace Walk Test as an Assessment of Mobility Limitation in Older Adults. J. Am. Geriatr. Soc..

[36] Chodzko-Zajko W.J., Proctor D.N., Fiatarone Singh M.A., Minson C.T., Nigg C.R., Salem G.J., Skinner J.S. (2009). American College of Sports Medicine position stand. Exercise and physical activity for older adults. Med. Sci. Sports Exerc..

[37] Pescatello L.S., Franklin B.A., Fagard R., Farquhar W.B., Kelley G.A., Ray C.A. (2004). American College of Sports Medicine position stand. Exercise and hypertension. Med. Sci. Sports Exerc..

[38] American College of Sports Medicine (2006). American College of Sports Medicine Guidelines for Exercise Testing and Prescription.

[39] Nelson M.E., Rejeski W.J., Blair S.N., Duncan P.W., Judge J.O., King A.C., Macera C.A., Castaneda-Sceppa C. (2007). Physical activity and public health in older adults: Recommendation from the American College of Sports Medicine and the American Heart Association. Med. Sci. Sports Exerc..

[40] Borg G. (1988). Perceived Exertion and Pain Scales.

[41] Jakicic J.M., Marcus M., Gallagher K.I., Randall C., Thomas E., Goss F.L., Robertson R.J. (2004). Evaluation of the Sense Wear Pro Armband to assess energy expenditure during exercise. Med. Sci. Sports Exerc..

[42] Cawthon P.M., Blackwell T.L., Cauley J.A., Ensrud K.E., Dam T.T., Harrison S.L., Peters K.W., Mackey D.C. (2013). Objective assessment of activity, energy expenditure, and functional limitations in older men: The osteoporotic fractures in men study. J. Gerontol.-Ser. A Biol. Sci. Med. Sci..

[43] Thabane L., Ma J., Chu R., Cheng J., Ismaila A., Rios L.P., Robson R., Thabane M., Giangregorio L., Goldsmith C.H. (2010). A tutorial on pilot studies: The what, why and how. BMC Med. Res. Methodol..

[44] Horne E., Lancaster G.A., Matson R., Cooper A., Ness A., Leary S. (2018). Pilot trials in physical activity journals: A review of reporting and editorial policy. Pilot Feasibility Stud..

[45] Eldridge S.M., Chan C.L., Campbell M.J., Bond C.M., Hopewell S., Thabane L., Lancaster G.A., O’Cathain A., Altman D., Bretz F. (2016). CONSORT 2010 statement: Extension to randomised pilot and feasibility trials. Pilot Feasibility Stud..

[46] Choo P.W., Rand C.S., Inui T.S., Lee M.L., Cain E., Cordeiro-Breault M., Canning C., Platt R. (1999). Validation of patient reports, automated pharmacy records, and pill counts with electronic monitoring of adherence to antihypertensive therapy. Med. Care.

[47] Cleveland J.C. (2010). Frailty, Aging, and Cardiac Surgery Outcomes: The Stopwatch Tells the Story. J. Am. Coll. Cardiol..

[48] Cruz-Jentoft A.J., Baeyens J.P., Bauer J.M., Boirie Y., Cederholm T., Landi F., Martin F.C., Michel J.P., Rolland Y., Schneider S.M. (2010). Sarcopenia: European consensus on definition and diagnosis. Age Ageing.

[49] Enright P.L., McBurnie M.A., Bittner V., Tracy R.P., McNamara R., Arnold A., Newman A.B. (2003). The 6-min walk test: A quick measure of functional status in elderly adults. Chest.

[50] Steele B. (1996). Timed walking tests of exercise capacity in chronic cardiopulmonary illness. J. Cardiopulm. Rehabil..

[51] O’Brien E., Waeber B., Parati G., Staessen J., Myers M.G. (2001). Clinical review Blood pressure measuring devices: Recommendations of the European Society of Hypertension. BMJ.

[52] Fitzmaurice G.M., Laird N.M., Ware J.H. (2012). Applied Longitudinal Analysis.

[53] Bates D., Mächler M., Bolker B.M., Walker S.C. (2014). Fitting linear mixed-effects models using lme4. arXiv.

[54] American College of Sports Medicine (2014). ACSM’s Guidelines for Exercise Testing and Prescription.

[55] Charlesworth C.J., Smit E., Lee D.S.H., Alramadhan F., Odden M.C. (2015). Polypharmacy Among Adults Aged 65 Years and Older in the United States: 1988–2010. J. Gerontol. Ser. A Biol. Sci. Med. Sci..

[56] Pahor M., Guralnik J.M., Ambrosius W.T., Blair S., Bonds D.E., Church T.S., Espeland M.A., Fielding R.A., Gill T.M., Groessl E.J. (2014). Effect of structured physical activity on prevention of major mobility disability in older adults: The LIFE Study randomized clinical trial. J. Am. Med. Dir. Assoc..

[57] Cesari M., Kritchevsky S.B., Atknson H.H., Penninx B.W., Bari M.D., Tracy R.P., Pahor M. (2009). Angiotensin converting enzyme inhibition and novel cardiovascular risk biomarkers. Am. Heart J..

[58] Perera S., Mody S.H., Woodman R.C., Studenski S.A. (2006). Meaningful change and responsiveness in common physical performance measures in older adults. J. Am. Geriatr. Soc..

[59] Anton S.D., Woodsa A.J., Ashizawac T., Barba D., Buford T.W., Carter C.S., Clark D.J., Cohen R.A., Corbett D.B., Cruz-Almeida Y. (2015). Successful Aging: Advancing the Science of Physical Independence in Older Adults. Ageing Res. Rev..

[60] Rajadhyaksha V. (2010). Conducting feasibilities in clinical trials: An investment to ensure a good study. Perspect. Clin. Res..

[61] El-Kotob R., Giangregorio L.M. (2018). Pilot and feasibility studies in exercise, physical activity, or rehabilitation research. Pilot Feasibility Stud..

[62] Katula J.A., Kritchevsky S.B., Guralnik J.M., Glynn N.W., Pruitt L., Wallace K., Walkup M.P., Hsu F.-C., Studenski S.A., Gill T.M. (2007). Lifestyle Interventions and Independence for Elders pilot study: Recruitment and baseline characteristics. J. Am. Geriatr. Soc..

[63] Whelton P.K., Carey R.M., Aronow W.S., Casey D.E., Collins K.J., Himmelfarb C.D., DePalma S.M., Gidding S., Jamerson K.A., Jones D.W. (2018). 2017 ACC/AHA/AAPA/ABC/ACPM/AGS/APhA/ASH/ASPC/NMA/PCNA Guideline for the Prevention, Detection, Evaluation, and Management of High Blood Pressure in Adults: Executive Summary: A Report of the American College of Cardiology/American Heart Association Task Force on Clinical Practice Guidelines. Circulation.

[64] Cryer M.J., Horani T., Dipette D.J. (2016). Diabetes and Hypertension: A Comparative Review of Current Guidelines. J. Clin. Hypertens..

[65] Cruz-Jentoft A.J., Landi F., Schneider S.M., Zúñiga C., Arai H., Boirie Y., Chen L.K., Fielding R.A., Martin F.C., Michel J. (2014). Prevalence of and interventions for sarcopenia in ageing adults: A systematic review. Report of the International Sarcopenia Initiative (EWGSOP and IWGS). Age Ageing.

[66] Liu S.-H., Eaton C.B., Driban J.B., McAlindon T.E., Lapane K.L. (2016). Comparison of self-report and objective measures of physical activity in US adults with osteoarthritis. Rheumatol. Int..

[67] Thyregod M., Bodtger U. (2016). Coherence between self-reported and objectively measured physical activity in patients with chronic obstructive lung disease: A systematic review. Int. J. COPD.

[68] Keidar S., Heinrich R., Kaplan M., Hayek T., Aviram M. (2001). Angiotensin II Administration to Atherosclerotic Mice Increases Macrophage Uptake of Oxidized LDL A Possible Role for Interleukin-6. Arter. Thromb. Vasc. Biol..

[69] Hayek T., Attias J., Coleman R., Brodsky S., Smith J., Breslow J.L., Keidar S. (1999). The angiotensin-converting enzyme inhibitor, fosinopril, and the angiotensin II receptor antagonist, losartan, inhibit LDL oxidation and attenuate atherosclerosis independent of lowering blood pressure in apolipoprotein E deficient mice. Cardiovasc. Res..

[70] Sivasubramaniam S., Kumarasamy B. (2017). Pleiotropic Effects of Losartan in Hypertensive Patients with Dyslipidemia. J. Clin. Diagn. Res..

[71] Srivastava A., Adams-huet B., Vega G.L., Toto R.D. (2016). Effect of losartan and spironolactone on triglyceride-rich lipoproteins in diabetic nephropathy. J. Investig. Med..

[72] Derosa G., Ragonesi P.D., Mugellini A., Ciccarelli L., Fogari R. (2004). Effects of telmisartan compared with eprosartan on blood pressure control, glucose metabolism and lipid profile in hypertensive, type 2 diabetic patients: A randomized, double-blind, placebo-controlled 12-month study. Hypertens. Res..

[73] Benson S.C., Pershadsingh H.A., Ho C.I., Chittiboyina A., Desai P., Pravenec M., Qi N., Wang J., Avery M.A., Kurtz T.W. (2004). Identification of telmisartan as a unique angiotensin II receptor antagonist with selective PPARgamma-modulating activity. Hypertens (Dallas, Tex. 1979).

[74] Morgan A.E., Mooney K.M., Wilkinson S.J., Pickles N.A., Mc Auley M.T. (2016). Cholesterol metabolism: A review of how ageing disrupts the biological mechanisms responsible for its regulation. Ageing Res. Rev..

[75] Scott D., Trbojevic T., Skinner E., Clark R.A., Levinger P. (2015). Associations of calf inter- and intra-muscular adipose tissue with cardiometabolic health and physical function in community-dwelling older adults. J. Musculoskelet. Neuronal Interact..

[76] Bennett J.A., Riegel B., Bittner V., Nichols J. (2002). Validity and reliability of the NYHA classes for measuring research outcomes in patients with cardiac disease. Heart Lung.

[77] Folstein M.F., Folstein S.E., McHugh P.R. (1975). “Mini-mental state”. A practical method for grading the cognitive state of patients for the clinician. J. Psychiatr. Res..

