# Supplementary material for: Multimodal Intervention to Improve Functional Status in Hypertensive Older Adults: A Pilot Randomized Controlled Trial
Source: J Clin Med. 2019 Feb 6;8(2):196. doi: 10.3390/jcm8020196 (PMC6406861; doi:10.3390/jcm8020196)
Supplement: Supplementary file 1 [file jcm-08-00196-s001.pdf]

Supplementary Table S1. Additional exclusion criteria.

Exclusion Criteria
Primary renal disease
Serum creatinine >2.5 mg/dL in men or >2.0 mg/dL in women
Serum potassium > 5.0 mEq/L
Urinary protein > 1 on dipstick
Abnormal liver enzymes
Severe cardiac disease (i.e., New York Heart Association class III or IV congestive heart failure [76])
Acute myocardial infarction identified by electrocardiogram
Significant cognitive impairment (defined as a known diagnosis or a Mini-Mental State Examination score <24 [77])
Inability to complete the 400-meter walk test within 15 minutes without sitting or interpersonal assistance (an indicator of disablement and likely inability to fully engage in the exercise intervention)
Regular participation in or absolute contraindications to exercise training according to the American College of Sport Medicine guidelines [38]
Other medical condition precluding safe participation
Simultaneous participation in another intervention trial

Supplementary Table S2. Change in clinical safety parameters from baseline to 24-week follow-up according to the randomization group.

Outcomes	Overall Baseline	Perindopril Adjusted Mean Change (95% CI) *	Losartan Adjusted Mean Change (95% CI) *	HCTZ Adjusted Mean Change (95% CI) *
Basic metabolic panel				
Sodium, mmol/L	139.9 (2.6)	−0.0 (−1.8, 1.7)	1.6 (0.1, 3.1)	−0.5 (−2.2, 1.3)
Potassium, mmol/L	4.25 (0.27)	0.3 (0.1, 0.6)	0.0 (−0.2, 0.3)	−0.3 (−0.5, −0.0)
Chloride, mmol/L	102.4 (2.5)	0.6 (−1.3, 2.6)	0.4 (−1.3, 2.1)	−1.8 (−3.7, 0.1)
Carbon Dioxide, mmol/L	22.8 (3.1)	−0.1 (−2.3, 2.0)	−0.7 (−2.7, 1.3)	1.6 (−0.6, 3.8)
Calcium, mg/dL	9.57 (0.40)	−0.1 (−0.3, 0.1)	−0.1 (−0.3, 0.1)	0.0 (−0.2, 0.3)
Total Protein, g/dL	7.11 (0.35)	−0.2 (−0.4, 0.1)	0.1 (−0.1, 0.4)	0.0 (−0.2, 0.3)
Albumin, g/dL	4.26 (0.23)	−0.1 (−0.2, 0.1)	0.0 (−0.1, 0.2)	−0.0 (−0.2, 0.1)
Globulin, g/dL	2.84 (0.33)	−0.1 (−0.2, 0.1)	0.1 (−0.1, 0.2)	−0.0 (−0.2, 0.2)
Albumin/Globulin Ratio	1.52 (0.20)	0.0 (−0.1, 0.1)	−0.0 (−0.1, 0.0)	−0.0 (−0.1, 0.1)
Bilirubin, mg/dL	0.63 (0.31)	−0.0 (−0.2, 0.1)	0.0 (−0.1, 0.2)	0.1 (0.0, 0.3)
Hepatic panel				
Alkaline Phosphatase, U/L	71.8 (23.2)	1.7 (−5.7, 9.1)	6.3 (−0.2, 12.8)	−1.4 (−8.9, 6.1)
AST, U/L	19.6 (4.1)	0.3 (−3.2, 3.7)	0.8 (−2.3, 3.9)	0.9 (−2.6, 4.3)
ALT, U/L	16.1 (3.2)	1.1 (−1.9, 4.2)	−0.0 (−2.7, 2.7)	0.2 (−2.8, 3.3)
Complete blood count				

White blood cell count, $10^3/\mu L$	6.20 (1.51)	−0.4 (−1.1, 0.3)	−0.3 (−0.9, 0.3)	0.1 (−0.6, 0.8)
Red blood cell count, $10^6/\mu L$	4.54 (0.46)	−0.1 (−0.2, 0.1)	0.0 (−0.1, 0.2)	−0.0 (−0.2, 0.1)
Hematocrit, %	44.5 (13.7)	4.6 (−0.5, 9.7)	3.4 (−1.1, 8.0)	4.0 (−1.1, 9.2)
Platelets count, $10^3/\mu L$	219.6 (49.1)	−12.6 (−42.3, 17.1)	2.3 (−24.2, 28.8)	8.6 (−21.4, 38.6)
Absolute Neutrophils, cell/μL	3756 (1439)	−157 (−758, 431)	110 (−411, 631)	7 (−591, 604)
Absolute Lymphocytes, cell/μL	1615 (521)	−100 (−351, 152)	22 (−203, 268)	72 (−192, 329)
Absolute Monocytes, cell/μL	448.6 (138.4)	−1.9 (−66.9, 63.1)	−5.1 (−62.0, 51.6)	−72.3 (−138.5, −6.1)
Absolute Eosinophils, cell/μL	163.4 (113.0)	−23.2 (−86.3, 39.8)	4.8 (−50.6, 60.2)	25.3 (−40.2, 90.7)
Absolute Basophils, cell/μL	32.5 (21.5)	−2.2 (−10.5, 6.0)	−7.4 (−14.8, −0.1)	6.4 (−1.9, 14.7)

Notes: ALT: Alanine Aminotransferase. AST: Aspartate aminotransferase. CI: Confidence Interval. Data are expressed as mean (SD). * Change within groups is presented as adjusted mean change with 95% confidence interval, adjusted to age, sex and baseline status. Reference range is according to limits established by the American College of Sports Medicine [55]
